# Options for improving low birthweight and prematurity birth outcomes of indigenous and culturally and linguistically diverse infants: a systematic review of the literature using the social-ecological model

**DOI:** 10.1186/s12884-021-04307-1

**Published:** 2022-01-03

**Authors:** Shae Karger, Claudia Bull, Joanne Enticott, Emily J. Callander

**Affiliations:** 1 School of Public Health and Preventive Medicine, Monash University, 553 St Kilda Rd, Melbourne, VIC 3004 Australia; 2 Monash Centre for Health Research and Implementation, Monash University, Melbourne, VIC Australia

**Keywords:** Birth outcomes, Systematic review, Indigenous, Culturally and linguistically diverse, CALD, First nations, Aboriginal, Health outcomes, Social-ecological model, Infant outcomes, Maternal outcomes

## Abstract

**Background:**

Prematurity and low birthweight are more prevalent among Indigenous and Culturally and Linguistically Diverse infants.

**Methods:**

To conduct a systematic review that used the social-ecological model to identify interventions for reducing low birthweight and prematurity among Indigenous or CALD infants. Scopus, PubMed, CINAHL, and Medline electronic databases were searched. Studies included those published in English between 2010 and 2021, conducted in high-income countries, and reported quantitative results from clinical trials, randomized controlled trials, case-control studies or cohort studies targeting a reduction in preterm birth or low birthweight among Indigenous or CALD infants. Studies were categorized according to the level of the social-ecological model they addressed.

**Findings:**

Nine studies were identified that met the inclusion criteria. Six of these studies reported interventions targeting the organizational level of the social-ecological model. Three studies targeted the policy, community, and interpersonal levels, respectively. Seven studies presented statistically significant reductions in preterm birth or low birthweight among Indigenous or CALD infants. These interventions targeted the policy (*n* = 1), community (*n* = 1), interpersonal (*n* = 1) and organizational (*n* = 4) levels of the social-ecological model.

**Interpretation:**

Few interventions across high-income countries target the improvement of low birthweight and prematurity birth outcomes among Indigenous or CALD infants. No level of the social-ecological model was found to be more effective than another for improving these outcomes.

**Supplementary Information:**

The online version contains supplementary material available at 10.1186/s12884-021-04307-1.

## Synopsis

A systematic review of the literature for improving birth outcomes of Indigenous and culturally and linguistically diverse infants using the social-ecological model.

## Introduction

Marginalised individuals in Australia, including Aboriginal, Torres-Strait Islander, First Nations (respectfully referred to as Indigenous Australians hereafter) and Culturally and Linguistically Diverse (CALD) women and infants have long had different experiences of health and healthcare in Australia compared to their non-marginalised counterparts. Indigenous infants make up approximately 5.2% of births each year in Australia (1 in 19 births) [1]. Low birthweight and prematurity are more prevalent among Indigenous infants compared with non-Indigenous infants [2]. Infants born to CALD women account for one-third of Australia’s births, and they too are among the most likely to experience low birthweight and prematurity [3]. Social determinants of health that differ for Indigenous Australians, including cultural identity, family support, participation in cultural activities and access to traditional lands, can contribute to differences in health outcomes such as low birthweight and prematurity, as well as quality of life within the Indigenous population [4].

Disadvantages and social determinants to CALD women that act as barriers to accessing care, and in turn contribute to poor birth outcomes include cultural differences, language barriers, limited health literacy, insufficient support, transport issues and limited financial capacity [3]. Additionally, marginalised women in Australia are at an increased risk of low birthweight and premature births secondary to factors that include intergenerational trauma, colonisation, and stigma and racism [5].

Extensive research has identified that individual-level risk factors for adverse pregnancy outcomes amongst Indigenous women may include smoking, excessive alcohol consumption, substance use, obesity, poor nutrition and gestational diabetes [6]. Additionally, there is little information regarding the individual level factors that may contribute to poor birth outcomes for CALD women, however the structural, organizational, and cultural barriers are evident [7]. There may be wider systematic factors that also contribute to these poorer outcomes, including low birthweight, and prematurity, for both Indigenous and CALD women.

The political structure of Australia’s healthcare system and the way healthcare is delivered and made available in Australia is not appropriate for all, particularly Indigenous and CALD women [8, 9]. Indigenous women identify that the delivery of health services in Australia is heavily underpinned by ‘white’ culture, which does not reflect the same values, beliefs and practices of Indigenous culture [9]. Further, current health policy and practices favor care that suppresses the voice of marginalized individuals, and identifies Indigenous people as the ‘problem’ [9]. This is a discourse that needs to be adjusted to recognise health system, social and policy factors that may also contribute to poorer health outcomes [9]. Such issues are likely to extend to CALD women and infants on the basis of race inequity [7]. Currently, inequities are attributed to socio-economic status or ethnicity, instead of the political choices about how to design, finance and deliver healthcare.

**Fig. 1:**
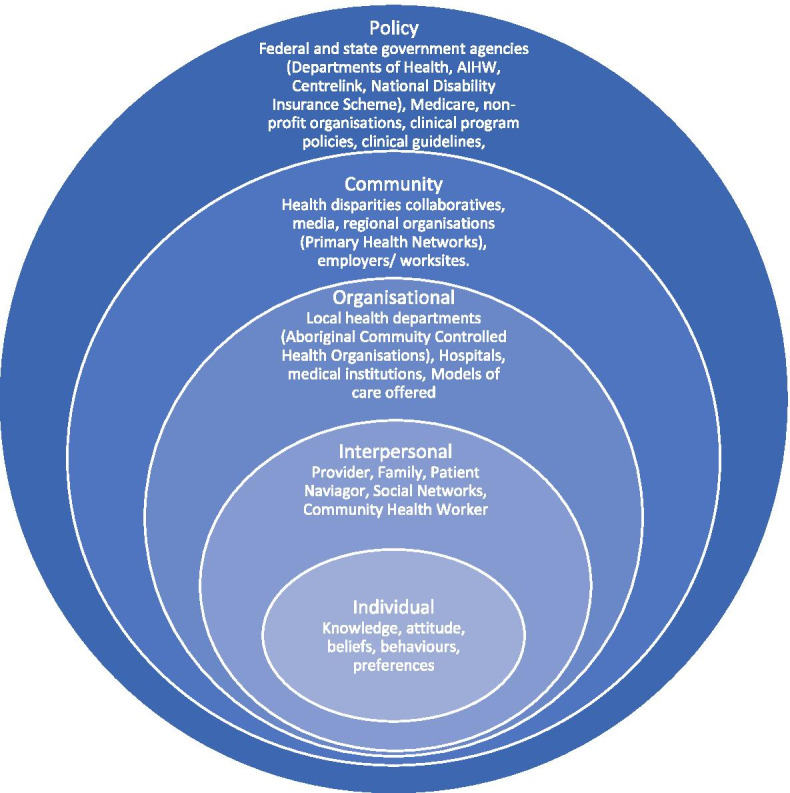
The Social-Ecological Model

The social-ecological model is a multi-level public health approach to prevention that considers broad social and political factors; not just individual ones [10]. The model consists of five levels, each encompassed within the next, beginning with the individual level, then interpersonal, organizational, community and policy levels, respectively (see Fig. 1). To obtain the greatest impact from public health interventions, it is recommended that interventions be applied at all levels of the model [10], as changes in broader levels are likely to impact on the levels nested within (e.g., individual factors such as smoking or alcohol consumption can be influenced by interpersonal, organizational, community and policy levels). Further, the social-ecological model has previously demonstrated impact for Indigenous Australians in interventions related to nutrition, physical activity, diabetes, men’s health, and substance use [11].

The Australian government recognizes that poorer individual level outcomes for Indigenous women are strongly associated with poorer socio-economic determinants [12]. However, disproportionate emphasis has been placed on the behavior of individuals, as opposed to wider societal and system factors that also contribute to poorer health outcomes [8]. Improvements made to Indigenous women’s access to antenatal care has resulted in an increase in the proportion of Indigenous mothers attending the first antenatal visit within 12 weeks of pregnancy, and an increase in the proportion of Indigenous mothers attending five or more antenatal visits [12]. There was also a decrease in the proportion of Indigenous mothers who reported smoking during pregnancy [12]. Despite these individual-level improvements, Indigenous mothers are still twice as likely to deliver infants of low birthweight compared to non-Indigenous mothers, and to deliver prematurely [2]. CALD women in Australia are also at an increased risk of delivering infants prematurely and with low birthweight, and it has been found that health service utilization by these women differs from those who are not marginalised [3].

Australia has a number of policies to improve the birth outcomes of women and infants in Australia, including some targeted specifically for Indigenous women and infants. The National Aboriginal and Torres Strait Islander Health Plan 2013–2023 is an evidence-based policy framework designed to guide policies and programs to improve Aboriginal and Torres Strait Islander Health [13]. Additionally, the National Maternity Services Plan aimed to provide culturally competent maternity care for Aboriginal and Torres Strait Islander women in an effort to reduce poor pregnancy and birth outcomes [14]. The plan identified three priority areas to improve services for Indigenous women; 1) developing and expanding culturally competent maternity care; 2) developing and supporting an Aboriginal workforce; and 3) developing dedicated programs for ‘Birthing on Country’ – best practice and culturally responsive maternal and infant healthcare for Indigenous women [15, 16]. Examples of culturally appropriate community-centred models of care include the Ngua Gundi Mother Child Project, Aboriginal Maternal and Infant Health Strategy (AMIHS), and Strong Women Strong Babies Strong Culture program [17]. In 2009, the report of the maternity services review for improving maternity services in Australia identified that Indigenous women have poorer maternal and perinatal outcomes. This report highlighted the need for culturally safe and community-centred models of care for these women. It is notable too that in Australia, there is an absence of policy focusing on reducing poor birth outcomes experienced by CALD women.

As such, there is an urgent need to address the poorer outcomes, particularly low birthweight, and prematurity, of infants born within marginalised groups living in Australia. The purpose of this systematic review was to identify interventions that aimed to reduce the incidence of preterm birth and low birthweight births in Indigenous and CALD mothers and infants, and examine which levels of the social-ecological model were addressed.

## Methods

The research questions guiding the systematic review were (1) What published interventions have aimed to improve preterm birth and low birthweight for Indigenous and Culturally and Linguistically Diverse women and infants; and (2) At what level of the social-ecological model do these interventions target?

Health status and socioeconomic disparities are prevalent in Australia, Canada, New Zealand and the United States between the Indigenous and non-Indigenous populations [18]. As these populations are similar and relevant to the population of Australia, literature from Canada, New Zealand and the United States, as well as Australia, will be included as well as any other high-income countries with comparable population groups. We will identify and synthesize evidence about types of interventions shown to be effective in reducing low birthweight, and prematurity, and to what extent interventions are aimed at the individual interpersonal, organizational, community or policy levels.

The review process was guided by the PRISMA guidelines for systematic reviews [19]. A systematic search of the literature was conducted through PubMed, SCOPUS, CINAHL, and Medline. Two different literature searches were undertaken, one each for Indigenous women and CALD women. All results were imported into one collection. For each search there were two groups of keywords indicative of low birthweight, and prematurity and either Indigenous terms or CALD terms (Appendix 1). The literature search was conducted in February 2021 and consulted again in April 2021.

**Fig. 2:**
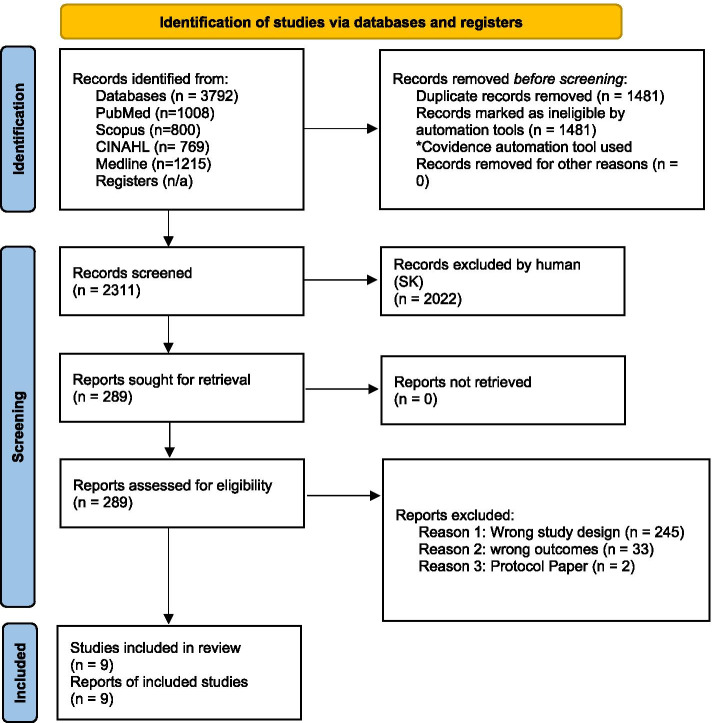
PRISMA 2020 flow diagram for new systematic reviews which included searches of databases and registers only

The studies were included in the review if they were a clinical or randomised controlled trial, case-control or cohort study, and had quantitative results. The search query was limited to articles in English, published in peer-reviewed journals within the last ten years (between 2010 and 2021), testing of an intervention or change, and presenting quantitative results. Search terms included “birth outcome*” “prematur*” “pregnan*” “birth weight” “birthweight” “preterm birth” and either “indigenous” “aborigin*” “first nation*” or “migrant” “refugee” “CALD” “culturally and linguistically diverse”. Articles that focused solely on descriptive studies of the poor birth outcome data, were protocols, or only reported qualitative results, were not included (refer to Fig. 2). Identifying high-income countries was not included as part of the search query, but was undertaken manually when assessing title, abstract and full text articles through Covidence.

The results from the searches of the four literature databases were imported into Covidence [20], and duplicates were initially removed. Abstract and full-text screening was performed, by one author (SK), with a random 25% checked by a second author (CB). Full-text screening was then performed by two authors (SK and EC). Article reference lists were hand-searched (by SK) for any relevant interventions that may have been missed during the literature search.

Data was extracted from the results of the included studies, including author, year of publication, country of intervention, study design, a description of the intervention, birth outcome (low birthweight or prematurity), whether it relates to Indigenous women or CALD women, and the effect size. The social-ecological model was the model utilised to categorise interventions, with the studies allocated based on what level of the social-ecological model they addressed – individual, organizational, community or policy level. Meta-analysis was deemed to be not appropriate, due to the high heterogeneity of included studies.

Narrative synthesis was used to integrate the findings of the included studies. Narrative synthesis collates the collective findings into a coherent, textual narrative, and is appropriate when the review question dictates the inclusion of a wide range of research designs, producing qualitative and/or quantitative findings for which other approaches to synthesis are inappropriate [21]. The included studies were categorised into their respective level of the social-ecological model based on which level the intervention targeted. This was done to identify if different levels of the social-ecological model were more effective than others at delivering interventions targeted at reducing low birthweight, and preterm term in marginalised women.

An assessment of quality of the included articles (*n* = 9) was conducted using the Joanna Briggs Institute (JBI) checklist for cohort studies [22]. Two of the authors (SK and EC) appraised the studies and no disagreements were identified. Studies that had a quality assessment score of 80% or above were included in the final review (*n* = 9). The score was achieved by calculating the number of boxes that ticked the YES column of the quality appraisal checklist for each study (Table 1).

**Table 1:**
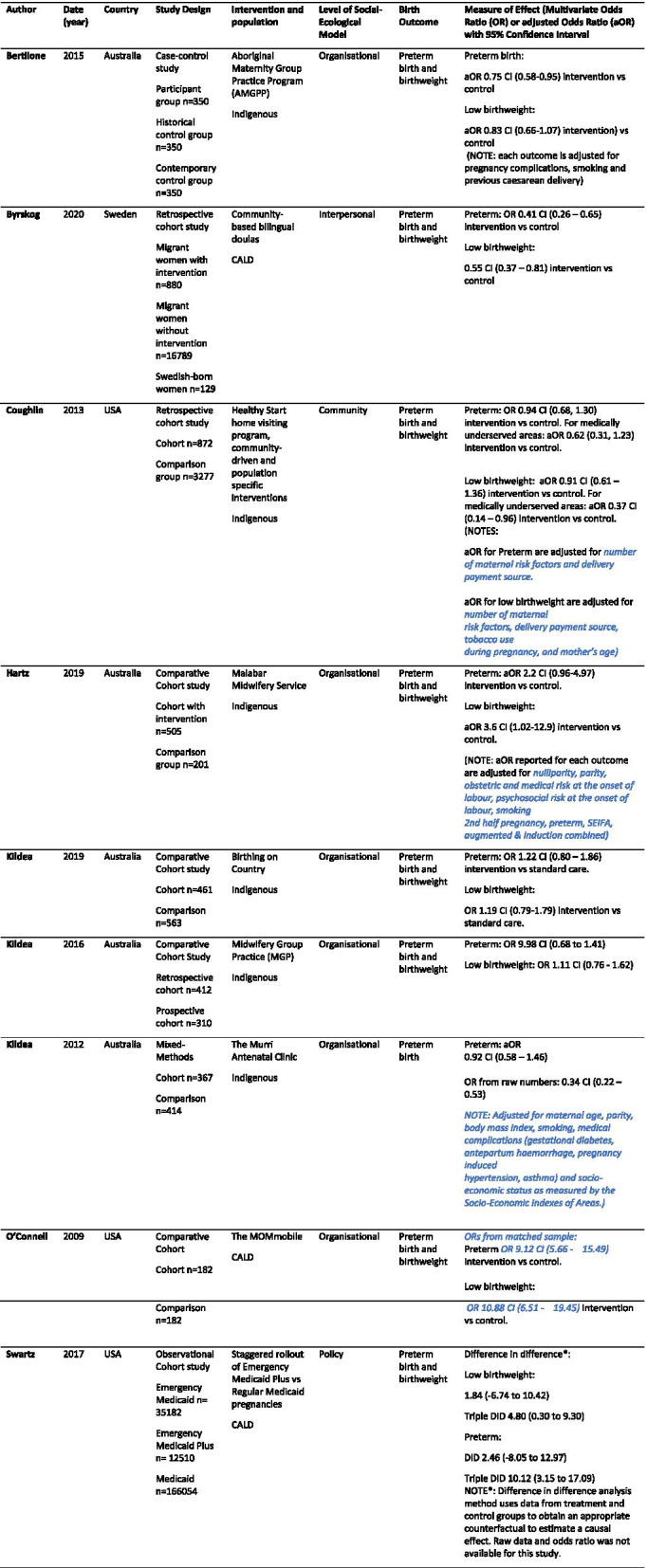
Overview of reviewed studies

## Results

Nine full-text articles met the reviews’ eligibility criteria (Table 2), and all met the quality appraisal criteria. In two studies there was some bias in recruitment of participants, but these studies appropriately adjusted results for confounding factors (Supplementary Information).

**Table 2:**
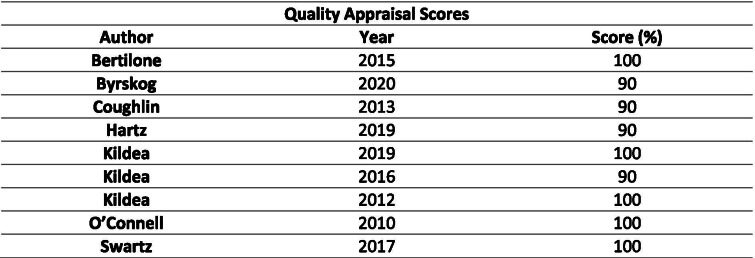
Quality Appraisal Scores for included papers

These studies described interventions for Indigenous (*n =* 6, 67%) and CALD women (*n* = 3, 33%) with the intention of improving or reducing low birthweight, and prematurityamong these populations. Eight of the studies were cohort studies, and one was a retrospective cohort study. Eight studies included low birth weight as an outcome (89%), seven studies including preterm birth as an outcome (78%), and six studies included both low birth weight and preterm birth as outcomes (67%). Studies were undertaken in Australia (*n* = 5, 56%), The United States of America (*n =* 3, 33%), and Sweden (*n* = 1, 11%).

**Fig. 3:**
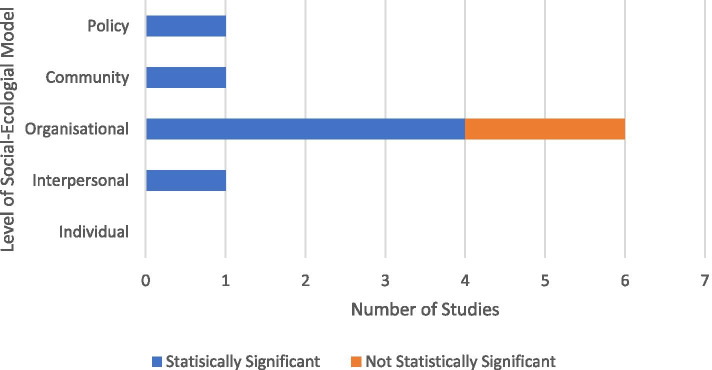
Statistical Significance of studies identified from the Systematic Review

The levels of the social-ecological model that the interventions targeted were interpersonal (*n =* 1, 11%), organisational (*n =* 6, 67%), community (*n =* 1, 11%), and policy (*n =* 1, 11%). Of the interventions included, five (56%) reported a statistically significant improvement in low birthweight and prematurity, and four (44%) reported no statistically significant improvement in prematurity and low birthweight through odds ratio and confidence intervals (CI). The single interventions aimed at interpersonal, community and policy outcomes all demonstrated an improvement in birth outcomes. Of the six interventions aimed at the organisational level, four (67%) demonstrated a significant improvement in birth outcomes (Fig. 3). The interpersonal, community and policy interventions all presented statistically significant effect measures. The community intervention (Coughlin, 2013) showed an odds ratio of 0.94 for reducing preterm birth and an odds ratio of 0.91 for reducing low birthweight. The odds ratio for the interpersonal intervention (Byrskog, 2020) showed an odds ratio of 0.41 for preterm birth and an odds ratio of 0.55 for low birthweight, which were both statistically significant. The effect measures for the policy intervention were presented in difference-in-difference ratios and were statistically significant for both preterm birth (DID = 2.46) and low birthweight (DID = 1.84). The organisational interventions [23, 24] both had odds ratios that were not statistically significant in reducing preterm birth and low birthweight. The intervention reported by Kildea, and colleague [23] had an odds ratio of 1.22 for preterm birth, and 1.19 for low birthweight. The intervention by O’Connell and colleagues [24] results showed preterm birth OR = 9.12 and low birthweight OR = 10.88, and was not statistically significant in reducing preterm birth and low birthweight.

## Discussion

The aim of this systematic review was to identify options for improving the low birthweight, and prematurity birth outcomes of Indigenous and CALD infants using the social-ecological model to classify intervention types. Overall, the results identified a limited number of interventions that targeted reducing preterm birth and low birthweight in infants of Indigenous and CALD women, though over half (seven of nine) produced statistically significant improvements in these outcomes [15, 16, 25–29]. The greatest improvements in outcomes were seen in interventions that targeted the organisational level of the social-ecological model. Greater research and evaluation of interventions targeting Indigenous and CALD women will be essential for actioning policies aimed at closing inequality gaps.

The interventions that were identified in this review that did provide evidence for improving outcomes took the form of an Aboriginal Maternity Group Practice Program (including Aboriginal grandmothers, Aboriginal Health officers and midwives) (Bertilone, 2015), the use of bilingual doulas [25], a multidisciplinary service alongside culturally appropriate midwifery care for Aboriginal and Torres Strait Islander mothers and infants [29], a home visiting program [26], an Aboriginal Community Controlled Health Organisation “Birthing in Our Community”, which also involved continuity of midwifery carer [23], a mobile medical van (MOMmobile) that provides on-site care before, during and after pregnancy to CALD women in Miami-Date Country in the United States [24], and expansion of universal healthcare policies [27].

Caseload midwifery has been identified as safe and cost-effective for women of any risk pregnancy [30] and the demand for continuity of care midwifery models of care for Indigenous women in Australia have been clear [31]. There is evidence to support the use of continuity of care models for midwifery care specifically for use for marginalised women such as Indigenous and CALD women. In addition to being highly valued and culturally safe, dedicated and integrated continuity of midwifery care with wraparound services for Indigenous mothers, is as safe as main stream services and promotes better clinical outcomes compared to national and state outcomes [29]. However, there were mixed results from this systematic review. One study demonstrated that midwifery group practice reduced preterm birth and low birthweight in the study sample [15], whereas another using caseload midwifery was effective at reducing preterm birth [23]. Thus, greater research is required to understand the benefits of and how best to implement midwifery-led models of maternity care for Indigenous and CALD women.

The interventions included in this review that targeted the organisational level of the social-ecological model [23, 24, 28] had a particular focus on ensuring that the care delivered was culturally appropriate. These results re-enforce the need to provide health services that are ethical, respectful and experienced as culturally safe. This may require a radical reconfiguration of the power distribution between Indigenous and non-Indigenous people for achieving better health outcomes, whereby Indigenous peoples could be considered the solution to better health rather than the cause of ill health [8, 9]. Therefore, any future interventions or studies intending to reduce health disparities involving marginalised women should be as ethical, respectful and as culturally safe as possible.

Birthing on country may be a key means of implementing the cultural appropriateness of care needed for Indigenous women in addition to reducing preterm birth and low birthweight [32]. Specifically, this reflects the need by Indigenous communities to have their infants born on the land [33]. Being born on country connects an Indigenous person to the land and community in a deeply cultural way, and provides life-long privileges and responsibilities for both the land and people [33]. Over a decade ago, it was accurately stated that for Indigenous women in Australia, birthing has moved *“from the personal to the political as governments provide policies about what is ‘best’ for Aboriginal women and their babies”* [33]. Thus, it is important for future research to consider the importance and benefits of ‘birthing on country’ for Indigenous women and their design. Additionally, Indigenous people have been involved in a global movement to return birthing services to Indigenous communities for over four decades [23].

The literature search for this review was undertaken with the assistance of a health librarian, thereby ensuring that the search terms used were comprehensive. Additionally, the quality appraisal and critique undertaken by authors demonstrated that the included studies were of high quality (scoring above 80%), suggesting that the conclusions from this review are robust.

Some of the included studies suggested selection bias in how women were recruited. Furthermore, due to the small number of studies included in this systematic review, and the spread of allocation across the social-ecological model, we were limited in concluding that one particular level of the social-ecological model was more effective at delivering interventions that reduce preterm birth and low birthweight than another particular level.

There were three articles identified in the full-text review stage of the systematic review that were protocols for proposed interventions on poor birth outcomes for women [34–36]. These protocols acknowledged the health disparities in birth outcomes between Indigenous women or CALD women, and non-Indigenous or CALD women in their respective countries and are currently ongoing studies. As such, they may present more evidence in the future.

Based on the outcomes of this review, it can be concluded that there is a general lack of evidence for what a good intervention for reducing low birthweight, and prematurity birth outcomes for marginalised women is. There is a need to conduct further research to trial interventions to improve infant outcomes for Indigenous and CALD women. Currently, there is little evidence for interventions that have shown to be effective. It is the recommendation of this review that there is not enough evidence to suggest that one particular level of the social-ecological model showed more statistically significant improvements than another, however whilst it is promising that interventions were found that assessed disparities in poor birth outcomes, there is a need to continue to invest in these areas of research.

## Supplementary Information

**Additional file 1.**

**Additional file 2.**

## Data Availability

All data generated or analysed during this study are included in this published article [and its supplementary information files].

## References

[1] Australian Institute of Health and Welfare (2018). Australia's mothers and babies 2016- in brief, in Perinatal statistics series 34.

[2] Australian Institute of Health and Welfare. Birthweight of babies born to indigenous mothers. Canberra; 2014.

[3] Rogers HJ (2020). Responding to the health needs of women from migrant and refugee backgrounds—models of maternity and postpartum care in high-income countries: a systematic scoping review. Health Soc Care Community.

[4] Australian Institute of Health and Welfare (2020). social determinants and indigenous health, in Australia's health 2020.

[5] Gibberd AJ (2019). A large proportion of poor birth outcomes among Aboriginal Western Australians are attributable to smoking, alcohol and substance misuse, and assault. BMC Pregnancy Childbirth.

[6] Gibson-Helm ME (2018). Identifying evidence-practice gaps and strategies for improvement in Aboriginal and Torres Strait islander maternal health care. PLoS One.

[7] Heslehurst N (2018). Perinatal health outcomes and care among asylum seekers and refugees: a systematic review of systematic reviews. BMC Med.

[8] Bond CJ, Singh D (2020). More than a refresh required for closing the gap of indigenous health inequality. Med J Aust.

[9] Durey A, Thompson SC (2012). Reducing the health disparities of indigenous Australians: time to change focus. BMC Health Serv Res.

[10] Centers for Disease Control and Prevention. Social Ecological Model. 2015 [cited 2020; Available from: http://medbox.iiab.me/modules/en-cdc/www.cdc.gov/cancer/crccp/sem.htm.

[11] Snijder M (2019). Developing an ecological framework of factors associated with substance use and related harms among Aboriginal and Torres Strait islander people: protocol for a systematic review. BMJ Open.

[12] Australian Institute of Health and Welfare (2020). Australia’s mothers and babies data visualisations.

[13] Australian Government (2013). National Aboriginal and Torres Strait islander health plan 2013–2023.

[14] Australian Government (2011). National Maternity Services Plan*,* 2011.

[15] Kildea S (2016). Remote links: redesigning maternity care for Aboriginal women from remote communities in northern Australia – a comparative cohort study. Midwifery.

[16] Kildea S, Magick Dennis F, Stapleton H, Birthing on country workshop report (2013). Australian Catholic University and Mater Medical Research Unit on behalf of the Maternity Services Inter-Jurisdictional Committee for the Australian Health Ministers' Advisory Council: Alice Springs.

[17] Australian Government (2009). Improving maternity health Services in Australia: the report of the maternity services review.

[18] Smylie J (2010). Indigenous birth outcomes in Australia, Canada, New Zealand and the United States - an overview. Open Womens Health J.

[19] Liberati A (2009). The PRISMA statement for reporting systematic reviews and Meta-analyses of studies that evaluate health care interventions: explanation and elaboration. PLoS Med.

[20] Covidence systematic review software. Melbourne: Veritas Health Innovation; Available at www.covidence.org.

[21] Aoki NJ, Enticott JC, Phillips LE (2013). Searching the literature: four simple steps. Transfusion.

[22] Joanna Briggs Institute (2021). Critical Appraisal Tools.

[23] Kildea S (2019). Reducing preterm birth amongst Aboriginal and Torres Strait islander babies: a prospective cohort study, Brisbane, Australia. EClinicalMedicine.

[24] O’Connell E (2010). Impact of a Mobile Van on prenatal care utilization and birth outcomes in Miami-Dade County. Matern Child Health J.

[25] Byrskog U, Small R, Schytt E (2020). Community-based bilingual doulas for migrant women in labour and birth – findings from a Swedish register-based cohort study. BMC Pregnancy Childbirth.

[26] Coughlin RL (2013). Pregnancy and birth outcome improvements for American Indians in the healthy start project of the inter-tribal Council of Michigan, 1998–2008. Matern Child Health J.

[27] Swartz JJ (2017). Expanding prenatal care to unauthorized immigrant women and the effects on infant health. Obstet Gynecol.

[28] Bertilone C, McEvoy S (2015). Success in closing the gap: favourable neonatal outcomes in a metropolitan Aboriginal maternity group practice program. Med J Aust.

[29] Hartz DL (2019). Evaluation of an Australian Aboriginal model of maternity care: the Malabar community midwifery link service. Women Birth.

[30] Tracy SK (2013). Caseload midwifery care versus standard maternity care for women of any risk: M@NGO, a randomised controlled trial. Lancet.

[31] West R, Gamble J, Kelly J, Milne T, Duffy E, Sidebotham M (2016). Culturally capable and culturally safe: Caseload care for Indigenous women by Indigenous midwifery students. Women Birth..

[32] Kildea S (2018). Birthing on country (in our community): a case study of engaging stakeholders and developing a best-practice indigenous maternity service in an urban setting. Aust Health Rev.

[33] Felton-Busch C (2009). Birthing on country: an elusive ideal?. Contemp Nurse.

[34] Kestler E (2013). A matched pair cluster randomized implementation trail to measure the effectiveness of an intervention package aiming to decrease perinatal mortality and increase institution-based obstetric care among indigenous women in Guatemala: study protocol. BMC Pregnancy Childbirth.

[35] Schytt E (2020). Community-based doula support for migrant women during labour and birth: study protocol for a randomised controlled trial in Stockholm, Sweden (NCT03461640). BMJ Open.

[36] Yelland J (2015). Bridging the gap: using an interrupted time series design to evaluate systems reform addressing refugee maternal and child health inequalities. Implement Sci.

