# Correction to: Options for improving low birthweight and prematurity birth outcomes of Indigenous and culturally and linguistically diverse infants: A systematic review of the literature using the social-ecological model

**DOI:** 10.1186/s12884-022-04599-x

**Published:** 2022-04-23

**Authors:** Shae Karger, Claudia Bull, Joanne Enticott, Emily Callander

**Affiliations:** 1 School of Public Health and Preventive Medicine, Monash University, 553 St Kilda Rd, Melbourne, VIC 3004 Australia; 2 Monash Centre for Health Research and Implementation, Monash University, Melbourne, VIC Australia

**Correction to: BMC Pregnancy Childbirth 22, 3 (2022)**

**https://doi.org/10.1186/s12884-021-04307-1**

Following publication of the original article [1], the authors reported an error in Table 1 noting that the OR reported by Kildea et al 2019 [2] is 0.50 (0.31-83), not 1.22 (0.80 – 1.86) as previously reported. This changes the result from not statistically significant, to statistically significant. This has been reflected in the final paragraph of the results section. Additionally, the authors noted that the paper Kildea et al 2012 [3] was not included in the reference list. This has been corrected and can be seen at reference #38. The reported Odds Ratio for this reference was also incorrect. The OR was 0.92 (0.59-1.43), and not 0.92 (0.58-1.46) as I reported. This has been corrected in the table. It does not change the results of the data or conclusions. Additionally, the measure of effect in Table 1 for Kildea et al 2016 [4] was generated from raw data in their paper. The authors have asked that we correct this to the previously published *P* Value of 0.906 for preterm and 0.122 for low birthweight. This has been corrected in table 1 and the corrections do not change the results of the data or the conclusions.
