# Supplementary material for: Options for improving low birthweight and prematurity birth outcomes of indigenous and culturally and linguistically diverse infants: a systematic review of the literature using the social-ecological model
Source: BMC Pregnancy Childbirth. 2022 Jan 3;22:3. doi: 10.1186/s12884-021-04307-1 (PMC8722221; doi:10.1186/s12884-021-04307-1)
Supplement: Supplementary file 1 — Additional file 1. [file 12884_2021_4307_MOESM1_ESM.pdf]

JBI Critical Appraisal Checklist for Cohort Studies

Reviewer____Shae Karger___________________Date__08/02/2021_____________

Author _____Bertilone______________________Year_2015__Record Number_____.

	Yes	No	Unclear	Not applicable
1. Were the two groups similar and recruited from the same population?	☑	☐	☐	☐
2. Were the exposures measured similarly to assign people to both exposed and unexposed groups?	☑	☐	☐	☐
3. Was the exposure measured in a valid and reliable way?	☑	☐	☐	☐
4. Were confounding factors identified?	☑	☐	☐	☐
5. Were strategies to deal with confounding factors stated?	☑	☐	☐	☐
6. Were the groups/participants free of the outcome at the start of the study (or at the moment of exposure)?	☑	☐	☐	☐
7. Were the outcomes measured in a valid and reliable way?	☑	☐	☐	☐
8. Was the follow up time reported and sufficient to be long enough for outcomes to occur?	☑	☐	☐	☐
9. Was follow up complete, and if not, were the reasons to loss to follow up described and explored?	☑	☐	☐	☐
10. Were strategies to address incomplete follow up utilized?	☑	☐	☐	☐
11. Was appropriate statistical analysis used?	☑	☐	☐	☐

Overall appraisal: Include ☑ Exclude ☐ Seek further info ☐

Comments (Including reason for exclusion)

JBI Critical Appraisal Checklist for Cohort Studies

Reviewer ____Shae Karger________________ Date ___08/02/2021___________

Author _____Byrskog___________________ Year _2020___ Record Number _____

	Yes	No	Unclear	Not applicable
1. Were the two groups similar and recruited from the same population?	☑	☐	☐	☐
2. Were the exposures measured similarly to assign people to both exposed and unexposed groups?	☐	☑	☐	☐
3. Was the exposure measured in a valid and reliable way?	☑	☐	☐	☐
4. Were confounding factors identified?	☑	☐	☐	☐
5. Were strategies to deal with confounding factors stated?	☑	☐	☐	☐
6. Were the groups/participants free of the outcome at the start of the study (or at the moment of exposure)?	☑	☐	☐	☐
7. Were the outcomes measured in a valid and reliable way?	☑	☐	☐	☐
8. Was the follow up time reported and sufficient to be long enough for outcomes to occur?	☑	☐	☐	☐
9. Was follow up complete, and if not, were the reasons to loss to follow up described and explored?	☑	☐	☐	☐
10. Were strategies to address incomplete follow up utilized?	☑	☐	☐	☐
11. Was appropriate statistical analysis used?	☑	☐	☐	☐

Overall appraisal: Include ☑ Exclude ☐ Seek further info ☐

Comments (Including reason for exclusion)

___

JBI Critical Appraisal Checklist for Cohort Studies

Reviewer____Shae Karger________________Date___08/02/2021___________

Author _____Coughlin__________________Year_ 2013___Record Number_____.

	Yes	No	Unclear	Not applicable
1. Were the two groups similar and recruited from the same population?	☑	☐	☐	☐
2. Were the exposures measured similarly to assign people to both exposed and unexposed groups?	☐	☑	☐	☐
3. Was the exposure measured in a valid and reliable way?	☑	☐	☐	☐
4. Were confounding factors identified?	☑	☐	☐	☐
5. Were strategies to deal with confounding factors stated?	☑	☐	☐	☐
6. Were the groups/participants free of the outcome at the start of the study (or at the moment of exposure)?	☑	☐	☐	☐
7. Were the outcomes measured in a valid and reliable way?	☑	☐	☐	☐
8. Was the follow up time reported and sufficient to be long enough for outcomes to occur?	☑	☐	☐	☐
9. Was follow up complete, and if not, were the reasons to loss to follow up described and explored?	☑	☐	☐	☐
10. Were strategies to address incomplete follow up utilized?	☑	☐	☐	☐
11. Was appropriate statistical analysis used?	☑	☐	☐	☐

Overall appraisal: Include ☑ Exclude ☐ Seek further info ☐

Comments (Including reason for exclusion)

JBI Critical Appraisal Checklist for Cohort Studies

Reviewer ____Shae Karger________________ Date___08/02/2021___________

Author _____Hartz________________________ Year_2019___ Record Number_____

	Yes	No	Unclear	Not applicable
1. Were the two groups similar and recruited from the same population?	☑	☐	☐	☐
2. Were the exposures measured similarly to assign people to both exposed and unexposed groups?	☐	☑	☐	☐
3. Was the exposure measured in a valid and reliable way?	☑	☐	☐	☐
4. Were confounding factors identified?	☑	☐	☐	☐
5. Were strategies to deal with confounding factors stated?	☑	☐	☐	☐
6. Were the groups/participants free of the outcome at the start of the study (or at the moment of exposure)?	☑	☐	☐	☐
7. Were the outcomes measured in a valid and reliable way?	☑	☐	☐	☐
8. Was the follow up time reported and sufficient to be long enough for outcomes to occur?	☑	☐	☐	☐
9. Was follow up complete, and if not, were the reasons to loss to follow up described and explored?	☑	☐	☐	☐
10. Were strategies to address incomplete follow up utilized?	☑	☐	☐	☐
11. Was appropriate statistical analysis used?	☑	☐	☐	☐

Overall appraisal: Include ☑ Exclude ☐ Seek further info ☐

Comments (Including reason for exclusion)

____

JBI Critical Appraisal Checklist for Cohort Studies

Reviewer Shae Karger Date 08/02/2021

Author Kildea Year 2019 Record Number

	Yes	No	Unclear	Not applicable
1. Were the two groups similar and recruited from the same population?	☑	☐	☐	☐
2. Were the exposures measured similarly to assign people to both exposed and unexposed groups?	☑	☐	☐	☐
3. Was the exposure measured in a valid and reliable way?	☑	☐	☐	☐
4. Were confounding factors identified?	☑	☐	☐	☐
5. Were strategies to deal with confounding factors stated?	☑	☐	☐	☐
6. Were the groups/participants free of the outcome at the start of the study (or at the moment of exposure)?	☑	☐	☐	☐
7. Were the outcomes measured in a valid and reliable way?	☑	☐	☐	☐
8. Was the follow up time reported and sufficient to be long enough for outcomes to occur?	☑	☐	☐	☐
9. Was follow up complete, and if not, were the reasons to loss to follow up described and explored?	☑	☐	☐	☐
10. Were strategies to address incomplete follow up utilized?	☑	☐	☐	☐
11. Was appropriate statistical analysis used?	☑	☐	☐	☐

Overall appraisal: Include ☑ Exclude ☐ Seek further info ☐

Comments (Including reason for exclusion)

JBI Critical Appraisal Checklist for Cohort Studies

Reviewer____Shae Karger________________Date___08/02/2021___________

Author _____Kildea______________________Year_ 2016__Record Number_____

	Yes	No	Unclear	Not applicable
1. Were the two groups similar and recruited from the same population?	☑	☐	☐	☐
2. Were the exposures measured similarly to assign people to both exposed and unexposed groups?	☑	☐	☐	☐
3. Was the exposure measured in a valid and reliable way?	☑	☐	☐	☐
4. Were confounding factors identified?	☑	☐	☐	☐
5. Were strategies to deal with confounding factors stated?	☐	☑	☐	☐
6. Were the groups/participants free of the outcome at the start of the study (or at the moment of exposure)?	☑	☐	☐	☐
7. Were the outcomes measured in a valid and reliable way?	☑	☐	☐	☐
8. Was the follow up time reported and sufficient to be long enough for outcomes to occur?	☑	☐	☐	☐
9. Was follow up complete, and if not, were the reasons to loss to follow up described and explored?	☑	☐	☐	☐
10. Were strategies to address incomplete follow up utilized?	☑	☐	☐	☐
11. Was appropriate statistical analysis used?	☑	☐	☐	☐

Overall appraisal: Include ☑ Exclude ☐ Seek further info ☐

Comments (Including reason for exclusion)

____

JBI Critical Appraisal Checklist for Cohort Studies

Reviewer____Shae Karger_________________Date___08/02/2021____________

Author _____Kildea_______________________Year_2012___Record Number______

	Yes	No	Unclear	Not applicable
1. Were the two groups similar and recruited from the same population?	☑	☐	☐	☐
2. Were the exposures measured similarly to assign people to both exposed and unexposed groups?	☑	☐	☐	☐
3. Was the exposure measured in a valid and reliable way?	☑	☐	☐	☐
4. Were confounding factors identified?	☑	☐	☐	☐
5. Were strategies to deal with confounding factors stated?	☑	☐	☐	☐
6. Were the groups/participants free of the outcome at the start of the study (or at the moment of exposure)?	☑	☐	☐	☐
7. Were the outcomes measured in a valid and reliable way?	☑	☐	☐	☐
8. Was the follow up time reported and sufficient to be long enough for outcomes to occur?	☑	☐	☐	☐
9. Was follow up complete, and if not, were the reasons to loss to follow up described and explored?	☑	☐	☐	☐
10. Were strategies to address incomplete follow up utilized?	☑	☐	☐	☐
11. Was appropriate statistical analysis used?	☑	☐	☐	☐

Overall appraisal: Include ☑ Exclude ☐ Seek further info ☐

Comments (Including reason for exclusion)

JBI Critical Appraisal Checklist for Cohort Studies

Reviewer Shae Karger Date 08/02/2021

Author O'Connell Year 2010 Record Number _____

	Yes	No	Unclear	Not applicable
1. Were the two groups similar and recruited from the same population?	☑	☐	☐	☐
2. Were the exposures measured similarly to assign people to both exposed and unexposed groups?	☑	☐	☐	☐
3. Was the exposure measured in a valid and reliable way?	☑	☐	☐	☐
4. Were confounding factors identified?	☑	☐	☐	☐
5. Were strategies to deal with confounding factors stated?	☑	☐	☐	☐
6. Were the groups/participants free of the outcome at the start of the study (or at the moment of exposure)?	☑	☐	☐	☐
7. Were the outcomes measured in a valid and reliable way?	☑	☐	☐	☐
8. Was the follow up time reported and sufficient to be long enough for outcomes to occur?	☑	☐	☐	☐
9. Was follow up complete, and if not, were the reasons to loss to follow up described and explored?	☑	☐	☐	☐
10. Were strategies to address incomplete follow up utilized?	☑	☐	☐	☐
11. Was appropriate statistical analysis used?	☑	☐	☐	☐

Overall appraisal: Include ☑ Exclude ☐ Seek further info ☐

Comments (Including reason for exclusion)

JBI Critical Appraisal Checklist for Cohort Studies

Reviewer___Shae Karger_______________Date___08/02/2021___________

Author _____Swartz____________________Year_ 2017___Record Number_____

	Yes	No	Unclear	Not applicable
1. Were the two groups similar and recruited from the same population?	☑	☐	☐	☐
2. Were the exposures measured similarly to assign people to both exposed and unexposed groups?	☑	☐	☐	☐
3. Was the exposure measured in a valid and reliable way?	☑	☐	☐	☐
4. Were confounding factors identified?	☑	☐	☐	☐
5. Were strategies to deal with confounding factors stated?	☑	☐	☐	☐
6. Were the groups/participants free of the outcome at the start of the study (or at the moment of exposure)?	☑	☐	☐	☐
7. Were the outcomes measured in a valid and reliable way?	☑	☐	☐	☐
8. Was the follow up time reported and sufficient to be long enough for outcomes to occur?	☑	☐	☐	☐
9. Was follow up complete, and if not, were the reasons to loss to follow up described and explored?	☑	☐	☐	☐
10. Were strategies to address incomplete follow up utilized?	☑	☐	☐	☐
11. Was appropriate statistical analysis used?	☑	☐	☐	☐

Overall appraisal: Include ☑ Exclude ☐ Seek further info ☐

Comments (Including reason for exclusion)

____
