# Supplementary material for: Options for improving low birthweight and prematurity birth outcomes of indigenous and culturally and linguistically diverse infants: a systematic review of the literature using the social-ecological model
Source: BMC Pregnancy Childbirth. 2022 Jan 3;22:3. doi: 10.1186/s12884-021-04307-1 (PMC8722221; doi:10.1186/s12884-021-04307-1)
Supplement: Supplementary file 2 — Additional file 2. [file 12884_2021_4307_MOESM2_ESM.pdf]

Literature Search Strategy

Pubmed CALD Search Strategy (216 results)

(((((refugee) OR (migrant*)) OR (asylum seeker)) AND ((((((((birthweight) OR (preterm birth)) OR (pre-term birth)) OR (neonatal weight)) OR ("birth weight"[MeSH Terms])) OR (infant, low birth weight[MeSH Terms])) OR ("birth outcome*")) OR (birth outcome*))) AND (intervention)) AND (study)
+ limits for [English] [published between December 2009-January 2020]

Pubmed Indigenous Search Strategy (792 results)

(((((((indigenous people*[MeSH Terms]) OR (indigenous)) OR ("first nation*")) OR ("first nation people*")) OR (aboriginal))) AND ((((((((((birthweight) OR (preterm birth)) OR (prematur*)) OR (pre-term birth)) OR (neonatal weight)) OR ("birth weight"[MeSH Terms])) OR (infant, low birth weight[MeSH Terms])) OR (birth outcome)) OR (birth outcome*)) OR ("birth outcome*"))) AND ((study) OR (intervention))
+ limits for [English] [published between December 2009-January 2020]

SCOPUS CALD search strategy (340 results)

((TITLE-ABS-KEY ("birth weight" OR birthweight OR "birth outcome*" OR "preterm birth" OR prematur* OR "pre-term birth" OR "neonatal weight")) AND (TITLE-ABS-KEY (refugee OR migrant* OR "asylum seeker"))) AND (TITLE-ABS-KEY (intervention OR study OR trial)) AND (LIMIT-TO (PUBYEAR , 2020) OR LIMIT-TO (PUBYEAR , 2019) OR LIMIT-TO (PUBYEAR , 2018) OR LIMIT-TO (PUBYEAR , 2017) OR LIMIT-TO (PUBYEAR , 2016) OR LIMIT-TO (PUBYEAR , 2015) OR LIMIT-TO (PUBYEAR , 2014) OR LIMIT-TO (PUBYEAR , 2013) OR LIMIT-TO (PUBYEAR , 2012) OR LIMIT-TO (PUBYEAR , 2011) OR LIMIT-TO (PUBYEAR , 2010) OR LIMIT-TO (PUBYEAR , 2009)) AND (LIMIT-TO (LANGUAGE , "English"))

SCOPUS Indigenous search strategy (460 results)

((TITLE-ABS-KEY (aborigin* OR indigenous OR {first nation*})) AND (TITLE-ABS-KEY ("birth weight" OR birthweight OR "birth outcome*" OR "preterm birth" OR prematur* OR "pre-term birth" OR "neonatal weight"))) AND (TITLE-ABS-KEY (intervention OR study OR trial)) AND (LIMIT-TO (PUBYEAR , 2020) OR LIMIT-TO (PUBYEAR , 2019) OR LIMIT-TO (PUBYEAR , 2018) OR LIMIT-TO (PUBYEAR , 2017) OR LIMIT-TO (PUBYEAR , 2016) OR LIMIT-TO (PUBYEAR , 2015) OR LIMIT-TO (PUBYEAR , 2014) OR LIMIT-TO (PUBYEAR , 2013) OR LIMIT-TO (PUBYEAR , 2012) OR LIMIT-TO (PUBYEAR , 2011) OR LIMIT-TO (PUBYEAR , 2010) OR LIMIT-TO (PUBYEAR , 2009)) AND (LIMIT-TO (LANGUAGE , "English"))

CINAHL CALD search strategy (336 results)

refugees or asylum seekers or displaced or migrants or immigrants or emmigration
AND
birthweight OR (birth weight or birth size or fetal weight) OR (preterm birth or premature birth or preterm labor or preterm delivery or prematurity) OR "birth weight" OR birth outcome racial disparities OR (birth outcomes or pregnancy outcomes or delivery outcomes or vaginal birth) OR neonatal weight
AND
Intervention
Study or trial
+ limits [English] [published between December 2009-January 2020]

CINAHL Indigenous search strategy (433 results)

indigenous or native or aboriginal or first nations or inuit or metis) OR indigenous peoples OR indigenous women OR aborigin*
AND
birthweight OR (birth weight or birth size or fetal weight) OR (preterm birth or premature birth or preterm labor or preterm delivery or prematurity) OR "birth weight" OR birth outcome racial disparities OR (birth outcomes or pregnancy outcomes or delivery outcomes or vaginal birth) OR neonatal weight
AND
Intervention
Study or trial
+ limits [English] [published between December 2009- January 2020]

Medline CALD search strategy (497 results)

refugees or asylum seekers or displaced or migrants or immigrants or emmigration
AND
birthweight OR (birth weight or birth size or fetal weight) OR (preterm birth or premature birth or preterm labor or preterm delivery or prematurity) OR "birth weight" OR birth outcome racial disparities OR (birth outcomes or pregnancy outcomes or delivery outcomes or vaginal birth) OR neonatal weight
AND
Intervention
Study or trial
+ limits [English] [published between December 2009-January 2020]

Medline Indigenous search strategy (718 results)

indigenous or native or aboriginal or first nations or inuit or metis) OR indigenous peoples OR indigenous women OR aborigin*
AND
birthweight OR (birth weight or birth size or fetal weight) OR (preterm birth or premature birth or preterm labor or preterm delivery or prematurity) OR "birth weight" OR birth outcome racial disparities OR (birth outcomes or pregnancy outcomes or delivery outcomes or vaginal birth) OR neonatal weight
AND
Intervention
Study or trial
+ limits [English] [published between December 2009-January 2020]
